# The switch between cataract surgical settings: Evidence from a time series analysis across 20 EU countries

**DOI:** 10.1371/journal.pone.0192620

**Published:** 2018-02-28

**Authors:** Maria Michela Gianino, Jacopo Lenzi, Marco Bonaudo, Maria Pia Fantini, Roberta Siliquini, Walter Ricciardi, Gianfranco Damiani

**Affiliations:** 1 Department of Public Health Sciences and Pediatrics, Università di Torino, Turin, Italy; 2 Department of Biomedical and Neuromotor Sciences, Alma Mater Studiorum–Università di Bologna, Bologna, Italy; 3 Department of Public Health, Università Cattolica del Sacro Cuore, Rome, Italy; Save Sight Institute, AUSTRALIA

## Abstract

**Objectives:**

To analyze trajectories of cataract surgery rates and to confirm the switch between inpatient cases and day surgery or outpatient cases.

**Design:**

Pooled, cross-sectional, time series analysis.

**Methods:**

Data on 20 European countries from 2004 to 2014 retrieved from the OECD.

**Results:**

The number of cataract surgery cases per 100,000 population has increased since 2004 (*b* = 31.1, *p* < 0.001, 95% CI = 26.7, 35.6). A reversal of the inpatient cases and same-day cases was found: the first ones decreased (*b* = –14.7, *p* < 0.001, 95% CI = –17.7, –11.8) while day surgery and outpatient cases increased (*b* = 37.5, *p* < 0.001, 95% CI = 31.6, 43.4, and *b* = 8.3, *p* = 0.001, 95% CI = 3.6, 13.1, respectively). Since 2004, the ratio of day surgery and outpatient cases to inpatient cases has grown significantly (*b* = 3.3, *p* < 0.001, 95% CI = 2.5, 4.0), reaching a share of 31.7 in 2014. However, this slope of 3.3 was not constant and slowed over the years: from 4.5 per year during the first five years to 1.9 in the second five. No association was found between cataract surgery rate and two regressors: elderly people, and health care expenditure per capita.

**Conclusion:**

EU countries have preserved cataract surgery, and this preservation is probably affected by the switch from inpatient to same-day surgery, thanks to the decrease in the cost and equivalent clinical outcomes. However, the slope of the switch slowed over time. Consequently, health care systems must support this process of change especially through reforms in financial and organizational fields.

## Introduction

Cataracts are the leading cause of visual impairment worldwide [1]. A cataract is defined as a clouding of the eye’s natural lens and is caused by several factors, the most frequent of which is the natural ageing process.

Quality of life in patients with cataracts is significantly reduced due to impaired vision [2]. Because cataracts occur primarily in older age groups, the accompanying decrease in functional abilities may be wrongly attributed to age-related processes. The only effective treatment for cataracts is surgical intervention; indeed, neither pharmacological nor dietary interventions have been shown to stop cataract formation [3].

Cataract surgery consists of removing the lens of the eye because of the presence of cataracts that are partially or completely clouding the lens and replacing it with an artificial lens. The use of intra-ocular lenses is now universally accepted as the treatment of choice, giving immediate and better visual rehabilitation than aphakic correction with spectacles. Cataract surgery has strengths and benefits that have contributed to its inclusion in recent essential surgery lists [4,5] as well as in the proposed initial surgical package for universal health coverage [6].

According to Ramke, these strengths and benefits lie in the fact that cataract surgery is a cost-effective intervention that usually restores sight. It can also improve quality-of-life, time-use, and social status, and it positively impacts on poverty alleviation [7].

Cataract surgery provides a good example of a high-volume surgery that from a medical point of view should not normally require hospitalization, although there may be some exceptions (e.g., people requiring general anesthesia or with severe comorbidities) [8], and it can now be carried out effectively on a same-day basis. The operation has changed from an inpatient to a same-day surgery thanks to advances in medical technologies, particularly the diffusion of less-invasive surgical interventions and better anesthetics. These innovations have also improved patient safety and health outcomes for patients and have in many cases reduced the unit cost per intervention by shortening the length of stay in hospitals.

The goal of this study is to analyze trajectories of cataract surgery rates across 20 EU countries by discussing the following questions:

- How has the incidence of cataract surgery changed in 20 EU countries over the 11-year period 2004 to 2014?
- How has the cataract surgery rate switched from an operation involving an overnight stay in hospital (inpatient cases) to an operation performed mainly as day cases (defined as a patient admitted to the hospital and discharged the same day) or outpatient cases in hospitals or outside hospitals (without any formal admission and discharge)?
- What association can be found between cataract surgery rate and older people and economic parameters?

Previous studies have inspected the issue of same-day cataract surgery rates. However, these studies focused on rates of a single country [9,10] or analyzed differences in the rates of many countries or regions and determined the factors influencing them for a single year [11,12].

## Materials and methods

This study used a pooled cross-sectional time series analysis of secondary data for 20 European countries during the period 2004 to 2014. These countries and years were chosen based on the availability of data. The unit of analysis was each country in each year (country-year). The countries included in the study were the following: Austria, Belgium, Czech Republic, Denmark, Estonia, Finland, France, Germany, Hungary, Ireland, Italy, Luxembourg, Norway, Poland, Portugal, Slovakia, Slovenia, Spain, Sweden, and the United Kingdom.

We obtained official data from the Organization for Economic Co-operation and Development (OECD) and Eurostat. The indicators considered here are shown in Table 1, which illustrates the definition and source for each of them.

**Table 1:** Indicators, definitions and data sources.

#	Indicator	Definition	Source
1	Cataract surgery—Total cases	Number of cataract surgeries per 100,000 population	OECD Health Statistics 2016
2	Cataract surgery—Inpatient cases	Number of cataract surgeries performed as inpatient cases per 100,000 population	OECD Health Statistics 2016
3	Cataract surgery—Day cases	Number of cataract surgeries performed as day cases per 100,000 population	OECD Health Statistics 2016
4	Cataract surgery—Outpatient cases	Number of cataract surgeries performed as outpatient cases per 100,000 population	OECD Health Statistics 2016
5	Population aged 65 years and over	Population aged 65 years and over (%)	Eurostat Statistics
6	Total expenditure on health care	Total expenditure on health care ($ per inhabitant at constant prices 2005)	OECD Health Statistics 2016
7	Public expenditure on health care	Public expenditure on health care ($ per inhabitant at constant prices 2005)	OECD Health Statistics 2016

*Abbreviations*: OECD, Organization for Economic Co-operation and Development.

In this study, the term same-day surgery case refers to both day surgery and outpatient cases. The term outpatient surgery case refers to patients who are not formally admitted in hospital or in any other health care facility and who are given surgical procedures performed in outpatient departments in hospitals, in emergency departments or in outside hospitals (ambulatory sector). The term day surgery case refers to patients who are given invasive surgical treatment (elective surgeries only) that are carried out in a dedicated surgical unit or part of a hospital and that lead to discharge on the day of the operation.

### Statistical analyses

This study used a pooled cross-sectional time series design to assess the relationship between dependent and independent variables over an 11-year period. A pooled cross-sectional time series design is one in which variables for a number of different cross-sections are observed over a certain time span [13]. The dependent variables are the indicators 1 to 4, while the independent variables are the indicators 5 to 7.

We performed a fixed effects linear regression because an ordinary least squares regression does not yield proper estimates on data containing repeated measures, and the alternative (the random effects model) was found to be inappropriate for use with these data due to results obtained from performing a Hausman test [14]. An advantage of fixed effects models is that they control for time-invariant heterogeneity among countries. Examples of factors potentially captured in fixed effects models include the following: cultural and historical patterns that shape social institution and policy systems, value systems that affect citizens’ outlook on life and illness perceptions, and factors shaping health-seeking behaviors. We also controlled for the presence of exogenous time trends in both the dependent and independent variables (i.e., time-fixed effects) by adding dummies to the model for each of the study years except for the first year.

Indicator #4 was excluded from regression analysis because cataract surgeries were performed as outpatient cases in only 8 of the 20 countries during the study period; indicator #7 was excluded because total and public expenditure on health care were strongly correlated (Pearson’s *r* = 0.99). The relationship between the remaining dependent and independent variables was assessed separately, resulting in 6 distinct fixed effects models. This choice was driven primarily by concerns about model over-fitting and multi-collinearity. The significance of each independent variable was assessed using robust standard errors due to results obtained from performing a modified Wald test for group-wise heteroscedasticity in the regression residuals [15].

For all analyses, the significance level was established at *p* < 0.05, and list-wise deletion was used. All data were analyzed using the *Stata software package*, *version 13* (StataCorp. 2013, Stata Statistical Software: Release 13; StataCorp LP, College Station, TX, USA).

## Results

**Fig 1:**
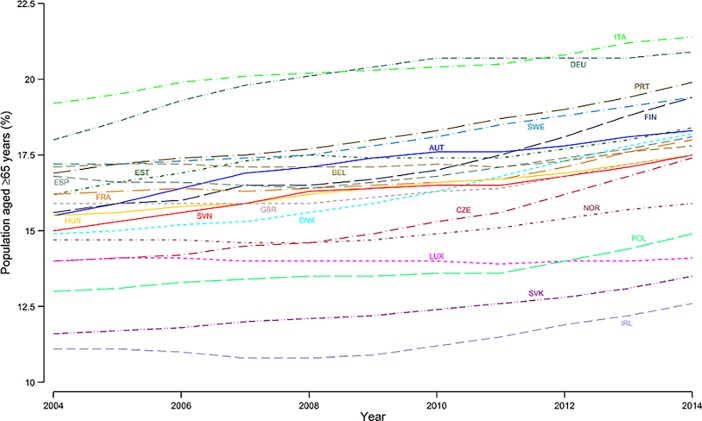
Individuals over 64 years of age (%) in 20 EU countries, year 2004 to 2014. *Abbreviations*: AUT, Austria; BEL, Belgium; CZE, Czech Republic; DEU, Germany; DNK, Denmark; ESP, Spain; EST, Estonia; FIN, Finland; FRA, France; GBR, United Kingdom; HUN, Hungary; IRL, Ireland; ITA, Italy; LUX, Luxembourg; NOR, Norway; POL, Poland; PRT, Portugal; SVK, Slovakia; SVN, Slovenia; SWE, Sweden.

In all 20 European countries, the proportion of individuals over 64 years increased. These results were confirmed in the fixed effects regression model (*b* = 0.20, *p* < 0.001, 95% CI = 0.18, 0.22). However, there are countries such as Italy where the proportion of elderly people was higher than 20%, as well as other countries, such as Ireland and Slovakia, where this proportion did not exceed 15% during the 11-year period (Fig 1).

**Fig 2:**
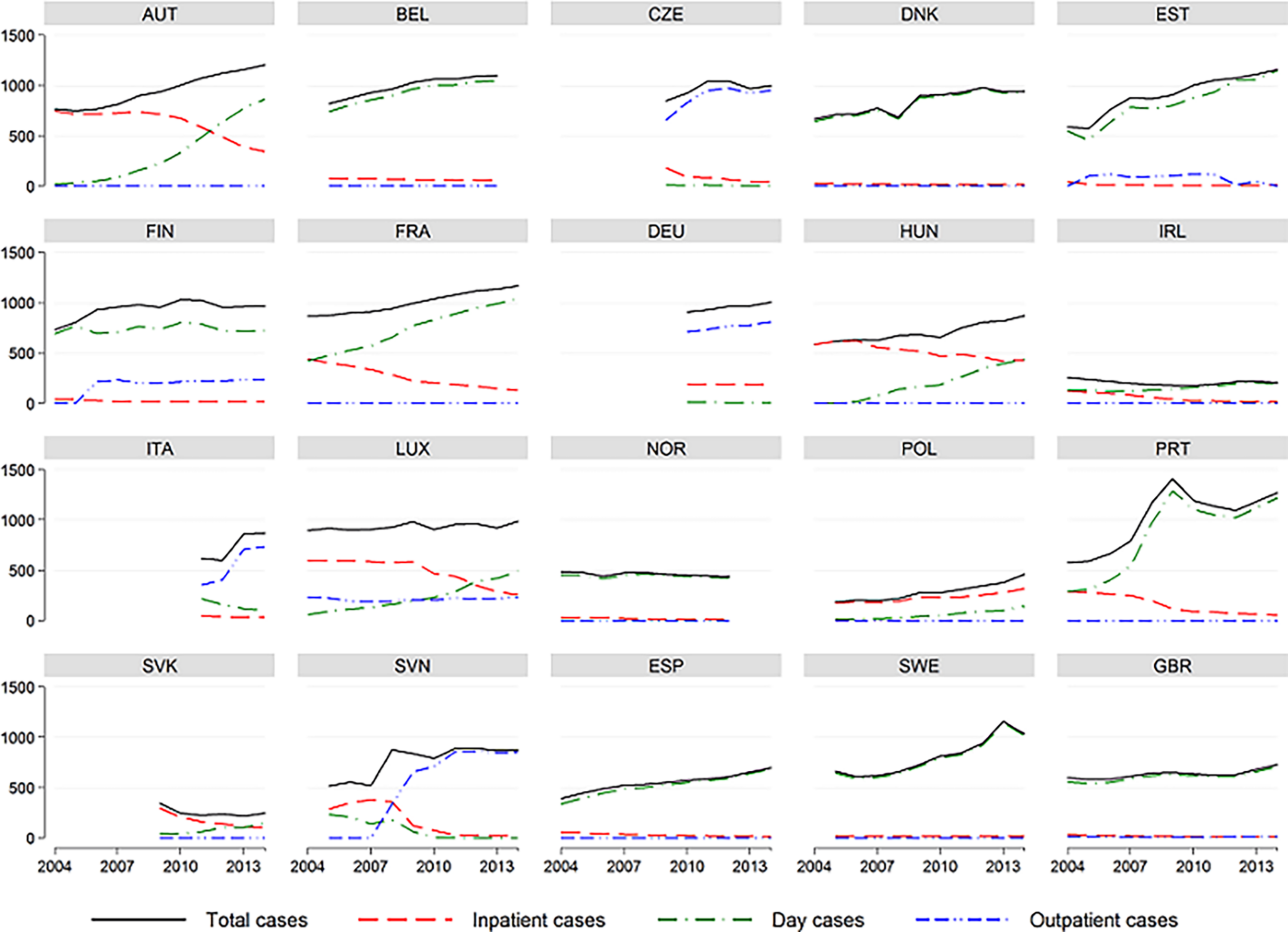
Total, inpatient, day and outpatient cases of cataract surgery (per 100,000) in 20 EU countries, years 2004 to 2014.

The number of cataract surgery cases per 100,000 population has increased since 2004 (Fig 2), although in Ireland, Norway and Slovakia, the number has decreased (*b* = 31.1, *p* < 0.001, 95% CI = 26.7, 35.6). The increase differs between countries, and in relative terms, the rate of intervention has grown more in 3 countries: Estonia, Poland and Portugal.

A reversal of the inpatient cases and same-day cases was found. Inpatient cases decreased (*b* = –14.7, *p* < 0.001, 95% CI = –17.7, –11.8), while day surgery cases increased (*b* = 37.5, *p* < 0.001, 95% CI = 31.6, 43.4) (Fig 2). Also, outpatient cases increased (*b* = 8.3, *p* = 0.001, 95% CI = 3.6, 13.1), although this setting is present in 8 countries out of 20 (Czech Republic, Estonia, Finland, Germany, Italy, Luxemburg, Slovenia, and the UK).

Fourteen states showed a switch with a progressive abandonment of cataract surgery in hospitalization, and they showed an increase of patients in day surgery. Italy, Germany, Slovenia and Czech Republic followed a path of reducing the rate of interventions on an inpatient basis to initially increase those in day surgery and then encourage those in outpatient settings. In Luxemburg, the switch with the reduction of interventions in ordinary hospitalization was compensated by an increase in DH and outpatient cases. Finland and Poland have shown a different behavior since 2004, because Finland had primarily focused on cataract surgery in DH and then delivered the patients in an outpatient setting, keeping always inpatient cases very low, while Poland continued to deliver them on an inpatient basis during the 11-year period analyzed.

**Fig 3:**
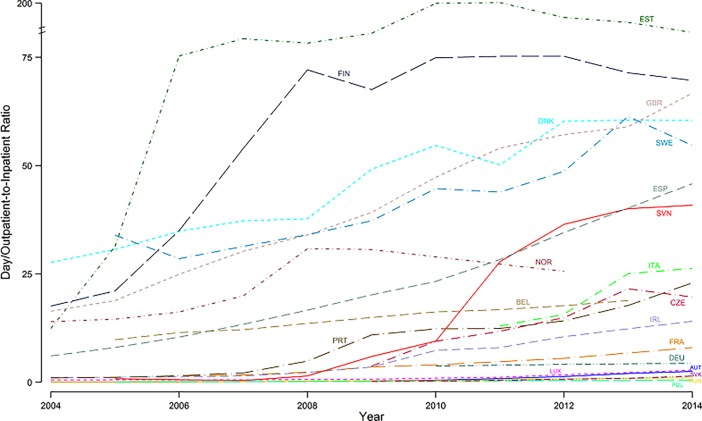
Ratio of day and outpatient cases to inpatient cases of cataract surgery in 20 EU countries, years 2004 to 2014.

Since 2004, the ratio of day and outpatient cases to inpatient cases has grown significantly (*b* = 3.3, *p* < 0.001, 95% CI = 2.5, 4.0), reaching a share of 31.7 in 2014. However, this slope of 3.3 was not constant and slowed over the years, as the significance and the sign of the quadratic term entered in the regression model demonstrates (*b* = –0.3, *p* = 0.043, 95% CI = –0.5, –0.01). Indeed, during the first five years, the average increase in the number of same-day surgery cases on the number of inpatient cases was equal to 3.9 per year, while in the second five it was equal to 1.0 per year. Time trends in the ratio of outpatient and day cases to inpatient cases for each EU country are presented in Fig 3.

The annual total health expenditure per capita (*b* = 141.9, *p* < 0.001, 95% CI = 132.3, 151.5) and annual public health expenditure per capita (*b* = 111.4, *p* < 0.001, 95% CI = 102.2, 120.5) increased during the 11-year period.

No association was found between cataract surgery rate and the regressors: elderly people, and health care expenditure per capita (Table 2).

**Table 2:** Results of regression analysis.

	Total cases	Inpatient cases	Day cases
Regressor	of cataract surgery	of cataract surgery	of cataract surgery
	Per 100,000	Per 100,000	Per 100,000
	inhabitants	inhabitants	inhabitants
% Population ≥ 65 years	71.4	29.6	10.2
	(38.8)	(29.1)	(27.5)
Time effect	3.89	4.87	4.43
	(<0.001)	(<0.001)	(<0.001)
*R*^*2*^	0.903	0.910	0.879
Health care expenditure per capita ($)	–0.06	0.04	–0.01
	(0.09)	(0.04)	(0.10)
Time effect	6.18	4.23	3.37
	(0.002)	(<0.001)	(0.472)
*R*^*2*^	0.897	0.910	0.879

*Notes*: Robust standard errors are given in parentheses under the coefficients, and *p*-values are given in parentheses under the *F*-statistics of the time effect. Total cases of cataract surgery include inpatient, day and outpatient cases.

## Discussion

This study investigated the change of incidence of cataract surgery in 20 EU countries over an 11-year period and the switch from operations involving an overnight stay in hospital (inpatient cases) to operations performed mainly as day cases (defined as a patient admitted to the hospital and discharged the same day) or outpatient cases in hospitals or outside hospitals (without any formal admission and discharge). Also, this study investigated the association between cataract surgery rate and older people and economic parameters.

There are a number of aspects of this study that must be considered before the implications of the findings can be discussed. The first limitation of this study is that differences observed in the switch and the number of inpatients, day surgery and outpatient cases may have been affected by differences in the available data and how cataract surgery cases are accounted for.

Criteria defining the requirements for cataract surgery were not analyzed. Consequently, the relationship between the need for and the supply of cataract extraction surgery was outside the scope of this paper.

Current health information systems in several countries remain incomplete in their coverage of same-day surgeries, especially those carried out in settings outside hospitals, and the absence or insufficiency of data from the private sector may partly explain the different levels of increase.

In the 11-year period examined, there has been a switch from inpatient surgery to surgical procedures carried out in an ambulatory setting or a day surgery setting (i.e., same-day) in EU countries, with the exception of Finland and Poland.

Indeed, our results also emphasize that the ratio of day and outpatient cases to inpatient cases has grown significantly.

A substantial amount of empirical evidence, demonstrating equivalent clinical outcomes between inpatient and same-day approaches [16–18] and advances in medical technologies, particularly the diffusion of less-invasive surgical interventions and better anesthetics, has made this switch possible [19].

Also, because of the relative scarcity of hospital beds in low-supply countries and the large reduction in some other countries, as a result of health care reforms, same-day surgery is now accepted as an alternative that can be used to meet the growing demand for health care. Since demand is increasing and inpatient capacity is decreasing, day surgery or outpatient surgery may serve as an alternative to meeting the demand. Of course, there is the question of causality. Does bed-supply decrease through the availability of alternatives such as same-day surgery, or is same-day surgery welcomed as a solution for decreasing inpatient capacity? We consider that same-day surgery acts as an alternative to decreasing the number of hospital beds, leading to causality running from bed supply to same-day surgery, since the hospital bed number was low or fell in EU countries even before same-day surgery became more commonplace. In addition, the hospital bed reductions often resulted from cost containment measures.

This switch may also be effected by economic benefits, especially in a global-budget situation, where the hospital receives a fixed amount. One of the main advantages of same-day surgery is the lower cost per case compared with inpatient surgery, due to shorter hospital stays, to a decrease in the time taken to perform surgical procedures and to better use of high-cost operating room apparatus and supplies.

Our results also emphasize that, during the switch, the growth in the number of same-day cases over the past 11 years exceeded the reduction in the number of cataract surgeries requiring an overnight stay in hospital. It is possible that the number of total cases has increased because the health care system expanded the services in geographic areas to meet local needs, such as in Estonia and Portugal. Also, this result may reflect an expansion effect [19] in surgical activities, depending on the reduction of the unit cost of such intervention and on the more productive potential of physicians by shortening the time of delivering procedures. Thus, many patients can be treated within the same working hours.

This suggestion may be supported by the fact that the countries showing an increase in the number of cataract procedures had already carried out by 2004 a high number of surgical procedures.

There is evidence that the switch is reaching a plateau in the EU countries. This slowdown may suggest that the health care systems cannot decrease the inpatients cases below a certain percentage, likely because a combination of barriers is going on, such as (i) a lack of high degree of regulations towards the less expensive same-day surgery that preclude the reimbursement for cases identified as inappropriate for inpatient surgery, and (ii) clinical practice traditions [11,20].

Also, this slowdown may suggest that the switch entails constructural and organizational changes in hospital and better primary care organization. Indeed, despite the fact that the hypotheses that a strong primary care organization and the existence of outpatient departments attached to hospitals would favor day surgery were not always confirmed by previous studies [20–23], it can be stated that same-day surgery requires good organization of and communication with home care (community nurses, general practitioners) and that constructional changes would be needed where no outpatient wards exist. Consequently, countries with a poor organization of home care and that are traditionally more oriented towards secondary care may face problems in implementing same-day surgery [20–23].

For some countries, such as the Slovak Republic, Austria and Hungary, where the share of same-day-surgery is still relatively low, the switch appeared to be slow. As reported by Gavurova and Soltes [24], some of the factors blocking further expansion of same-day surgery in Slovakia include geographical conditions, the social situation and the lowering prices by 30–50% compared to prices for regular finished hospitalizations. In Hungary the switch is slow especially because of national regulations; indeed the government just recently abolished the budget cap on the number of same-day surgery that can be performed in hospital [25].

In Austria, the slower switch may be explained by the fact that, until 2005, the health insurance did not pay for same-day surgery, and the hospitals did not get credits for day surgery. Meanwhile this has changed, so now hospitals get the same amount of credits for same-day as for inpatient surgery [11]. Another factor to explain the slower switch was the distance between surgeon/hospital and patient. In Austria the number of day-care units is still low and the distribution of ophthalmologists is very uneven. Most of them are concentrated in the cities, and very few services and contracts for specialists are offered in countryside areas; consequently, a long distance to hospitals and underdeveloped patient support, such as transportation services and hotels adjacent to the hospitals, influence the ratio of same-day cases [10]. Also, as reported by Kroneman [21], oversupply of inpatient beds still leads to a preference for inpatient care.

As mentioned above, in Poland no switch was made, and since 2009 inpatient cases have increased. The reason for this result may be at least threefold. Firstly, since July 2008 all hospitals (public and private) that have contracts with the National Health Fund must classify their patients using the Jednorodne Grupy Pacjentów (JGP) in order to receive DRG-based hospital payment for services they deliver, and inpatient cataract surgery is more advantageous than the prior system of payment. Secondly, in Poland there is a large number of non-public hospitals, which, by the end of 2007, had increased, while the number of public hospitals had decreased. Non-public hospitals had every incentive to deliver inpatient surgery. Thirdly, the average financial remuneration for health care personnel at state hospitals was rather low, which may have indirectly discouraged employees from increasing their surgical performance and may have limited the number of surgeries scheduled [26,27].

The switch has particular features in four EU countries (Italy, Germany, Czech Republic, Slovenia). Indeed, the switch is from inpatient surgery to surgical procedures carried out in an ambulatory setting.

In Italy, Czech Republic, and Slovenia, the switch includes an intermediate step, from inpatient surgery to day surgery. In Germany, the switch is directed from inpatient to outpatient surgery. A thrust in this direction may be given by the harmonization of the financial incentives with the process of change. This is a model being used by authorities, such as Italy or Slovenia, which has recently put in place regulations that will limit reimbursement for procedures identified as appropriate for day surgery.

Regression analysis shows that there is no significant association between the percentage of the elderly population and the number of cataract procedures per 100,000 population. This result suggests that patients are undergoing intervention at younger ages [9]. However, an increased percentage of elderly increases the number of cataract surgery procedures—this result could be explained by an improvement in healthy ageing. A further increase in the elderly increases the number of inpatient cases more so than same-day surgery cases, perhaps because these countries tend to concentrate inpatient services on the most serious cases.

To our knowledge there are very few studies that associate the health care expenditure per capita and the number of cataract procedures per 100,000 inhabitants in 20 EU countries, however it seems important to assess whether the total cases of cataracts and the switch depended on public or total health expenditure.

Our findings show that there is no significant association between the total health care expenditure per capita and the number of cataract procedures per 100,000 inhabitants. This result suggests that eye health is prioritized and this result is probably influenced by the fact that cataract surgery has an increasingly low cost thanks to the switch in same-day surgery. In addition, there is no significant association between the public health care expenditure per capita and the number of cataract procedures, and this result suggests that the private sector does not offset public sector.

In conclusion our study reveals the following: (i) the increasing trend of cataract cases was continuing to 2014, not only because of the expanding aging population; (ii) in the 11-year study period, there has been a switch from inpatient surgery to surgical procedures carried out in same-day setting. This switch was influenced by several factors that played with multiple weight in the 20 European countries. However, the literature and history of the countries studied highlight mainly the negative influence of acute-care bed and ophthalmologists density and the positive influence of financial incentives and regulations on the choice of surgeons. Also (iii), there was a slowdown in the switch, highlighting that the reversal of the inpatient cases and same day-cases must be supported over time.

From a medical point of view, a cataract surgery using modern techniques should not normally require a hospitalization, and inpatient surgery is indicated only in most serious cases requiring general anesthesia or with severe comorbidities[8]. This stresses the fact that the switch is primarily depending on non-medical reasons but on decision of health policy makers who must harmonize interventions to overcome the barriers within the countries.

## Supporting information

S1 Dataset Supporting data. (XLS)

## References

[1] Allen D, Vasavada A. Cataract and surgery for cataract. BMJ. 2006;333: 128–132. doi: 10.1136/bmj.333.7559.128 16840470 10.1136/bmj.333.7559.128 PMC1502210

[2] Heijl A, Leske MC. Cataract epidemiology. Ophthalmology. 2007;114: 201 doi: 10.1016/j.ophtha.2006.08.033 17198867 10.1016/j.ophtha.2006.08.033

[3] Chang DF. Tackling the greatest challenge in cataract surgery. Br J Ophthalmol. 2005;89: 1073–1074. doi: 10.1136/bjo.2005.068213 16113349 10.1136/bjo.2005.068213 PMC1772843

[4] Mock CN, Donkor P, Gawande A, Jamison DT, Kruk ME, Debas HT, et al Essential surgery: key messages from Disease Control Priorities, 3rd edition. Lancet Lond Engl. 2015;385: 2209–2219. doi: 10.1016/S0140-6736(15)60091-5 10.1016/S0140-6736(15)60091-5 PMC7004823 25662414

[5] Henry JA, Bem C, Grimes C, Borgstein E, Mkandawire N, Thomas WEG, et al Essential surgery: the way forward. World J Surg. 2015;39: 822–832. doi: 10.1007/s00268-014-2937-9 25566979 10.1007/s00268-014-2937-9

[6] Meara JG, Leather AJM, Hagander L, Alkire BC, Alonso N, Ameh EA, et al Global Surgery 2030: evidence and solutions for achieving health, welfare, and economic development. Lancet Lond Engl. 2015;386: 569–624. doi: 10.1016/S0140-6736(15)60160-X 10.1016/S0140-6736(15)60160-X 25924834

[7] Ramke J, Gilbert CE, Lee AC, Ackland P, Limburg H, Foster A. Effective cataract surgical coverage: An indicator for measuring quality-of-care in the context of Universal Health Coverage. PLOS ONE. 2017;12: e0172342 doi: 10.1371/journal.pone.0172342 28249047 10.1371/journal.pone.0172342 PMC5382971

[8] Lundström M, Barry P, Henry Y, Rosen P, Stenevi U. Evidence-based guidelines for cataract surgery: guidelines based on data in the European Registry of Quality Outcomes for Cataract and Refractive Surgery database. J Cataract Refract Surg. 2012;38: 1086–1093. doi: 10.1016/j.jcrs.2012.03.006 22541829 10.1016/j.jcrs.2012.03.006

[9] Keenan T, Rosen P, Yeates D, Goldacre M. Time trends and geographical variation in cataract surgery rates in England: study of surgical workload. Br J Ophthalmol. 2007;91: 901–904. doi: 10.1136/bjo.2006.108977 17540694 10.1136/bjo.2006.108977 PMC1955650

[10] Weingessel B, Richter-Mueksch S, Weingessel A, Gnad H, Vécsei-Marlovits PV. Is day-case cataract surgery an attractive alternative from the patients’ point of view? A questionnaire survey. Wien Klin Wochenschr. 2008;120: 756–760. doi: 10.1007/s00508-008-1113-3 19122987 10.1007/s00508-008-1113-3

[11] Mojon-Azzi SM, Mojon DS. The rate of outpatient cataract surgery in ten European countries: an analysis using data from the SHARE survey. Graefes Arch Clin Exp Ophthalmol Albrecht Von Graefes Arch Klin Exp Ophthalmol. 2007;245: 1041–1044. doi: 10.1007/s00417-007-0550-4 10.1007/s00417-007-0550-4 17318563

[12] Cillino S, Casuccio A, Di Pace F, Pillitteri F, Cillino G, Lodato G. Day care cataract surgery in Central and Southern Italy: a multicentric survey. BMC Health Serv Res. 2007;7: 16 doi: 10.1186/1472-6963-7-16 17270040 10.1186/1472-6963-7-16 PMC1797011

[13] Sayrs L. Pooled Time Series Analysis. Thousand Oaks, CA: Sage; 1989.

[14] Hsiao C. Analysis of Panel Data. Cambridge: Cambridge University Press; 1986.

[15] Greene WH. Econometric Analysis. 4th ed. New York: Prentice Hall; 2000.

[16] Castells X, Alonso J, Castilla M, Ribó C, Cots F, Antó JM. Outcomes and costs of outpatient and inpatient cataract surgery: a randomised clinical trial. J Clin Epidemiol. 2001;54: 23–29. 11165465 10.1016/s0895-4356(00)00271-7

[17] Fedorowicz Z, Lawrence D, Gutierrez P. Day care versus in-patient surgery for age-related cataract. Cochrane Database Syst Rev. 2005; CD004242 doi: 10.1002/14651858.CD004242.pub3 21735397 10.1002/14651858.CD004242.pub4

[18] Fedorowicz Z, Lawrence DJ, Gutierrez P. A Cochrane Systematic Review finds no significant difference in outcome or risk of postoperative complications between day care and in-patient cataract surgery. Saudi Med J. 2006;27: 1296–1301. 16951761

[19] Fattore G, Torbica A. Cost and reimbursement of cataract surgery in Europe: a cross-country comparison. Health Econ. 2008;17: S71–82. doi: 10.1002/hec.1324 18186033 10.1002/hec.1324

[20] Castoro C, Bertinato L, Baccaglini U, Drace CA, McKee M, Europe WHORO for, et al. Day surgery: making it happen. 2007; Available: http://www.who.int/iris/handle/10665/107831

[21] Kroneman MW, Westert GP, Groenewegen PP, Delnoij DMJ. International variations in availability and diffusion of alternatives to in-patient care in Europe: the case of day surgery. Ambul Surg. 2001;9: 147–154. doi: 10.1016/S0966-6532(01)00120-2

[22] Van der Zee J, Arnold M, Litsch M, Schwarz F. Deutschland-Niederlande 2:1 bei den Krankenhausaufnahmen-ist der Grund dazu im niederländische Hausärztesystem zu suchen? (Germany-Netherlands 2:1 in hospital admission rates-can this be explained from the Dutch GP system?). Krankenh-Report’99 Schwerpkt Versorg Chron Kranker Hosp Report’99 Theme Care Chronic Ill Stuttg N Y Schattauer. 2000; 203–212.

[23] Sorgatz H. Ambulantes Operieren und Tageschirurgy im Krankenhaus; Das Gesundheitsstrukturgesetz als gesundheitspolitische Vorgabe für ambulantes und tageschirurgisches operieren (Ambulatory treatment and day surgery in hospitals; The Health Care Structure law as health policy issue for ambulatory and day surgery). Zentralblatt Für Chir. 1994;119: 455–459. 7941791

[24] Gavurova B, Soltes M. System of day surgery in Slovakia: analysis of pediatric day surgery discrepancies in the regions and their importance in strategy of its development. 2016; doi: 10.15240/tul/001/2016-1-006

[25] OECD. Health at a Glance 2015 [Internet]. Paris: Organisation for Economic Co-operation and Development; 2015. Available: http://www.oecd-ilibrary.org/content/book/health_glance-2015-en

[26] Reinhard B, Alexander G, Wilm Q. Diagnosis-Related Groups In Europe: Moving Towards Transparency, Efficiency And Quality In Hospitals. McGraw-Hill Education (UK); 2011.

[27] Kocur I, Resnikoff S, Foster A. Eye healthcare services in eastern Europe: Part 1 Cataract surgery. Br J Ophthalmol. 2002;86: 847–850. 12140200 10.1136/bjo.86.8.847 PMC1771220

